# Reduced-dose nanoparticle albumin-bound-paclitaxel combined with ramucirumab as second-line treatment for advanced gastric cancer: a multicenter phase II study

**DOI:** 10.1186/s12885-026-16587-x

**Published:** 2026-07-25

**Authors:** Michihisa Iida, Chiyo Nakashima, Toru Kawaoka, Junya Kondo, Tsunenori Yamamoto, Shigefumi Yoshino, Kazuhisa Tokuno, Takayuki Kuga, Koji Dairaku, Yuki Nakagami, Yusaku Watanabe, Hiroto Matsui, Yoshitaro Shindo, Hidenori Takahashi, Tatsuya Ioka, Hiroaki Nagano

**Affiliations:** 1 Department of Gastroenterological, Breast and Endocrine Surgery, Yamaguchi University Graduate School of Medicine, 1-1-1 Minami-Kogushi, Ube, Yamaguchi 755-8505 Japan; 2 Department of Surgery, Tokuyama Central Hospital, Yamaguchi, Japan; 3 Department of Surgery, Ube Central Hospital, Yamaguchi, Japan; 4 Department of Surgery, NHO Kanmon Medical Center, Yamaguchi, Japan; 5 Department of Surgery, Tsushimi Hospital, Yamaguchi, Japan; 6 Department of Surgery, Nagato General Hospital, Yamaguchi, Japan; 7 Department of Surgery, Onoda Municipal Hospital, Yamaguchi, Japan; 8 Department of Data Science, Faculty of Data Science, Shimonoseki City University, Yamaguchi, Japan; 9 Oncology Center, Yamaguchi University Hospital, Yamaguchi, Japan

**Keywords:** Gastric cancer, Second-line chemotherapy, Nab-paclitaxel, Ramucirumab, Phase II trial

## Abstract

**Background:**

Weekly paclitaxel combined with ramucirumab is the standard second-line chemotherapy regimen for advanced gastric cancer. Nanoparticle albumin-bound paclitaxel (nab-paclitaxel) is increasingly used as it lowers the risk of hypersensitivity reactions; however, full-dose nab-paclitaxel (100 mg/m²) combined with ramucirumab frequently leads to severe myelosuppression. In clinical practice, dose reduction is often required, particularly in older patients or those with a poor performance status. However, the clinical efficacy and safety of a reduced-dose regimen of nab-paclitaxel combined with ramucirumab have not yet been prospectively evaluated. Accordingly, this multicenter phase II study aimed to assess the efficacy and tolerability of reduced-dose nab-paclitaxel combined with ramucirumab as second-line treatment for advanced gastric cancer.

**Methods:**

This open-label, single-arm, multicenter phase II trial was conducted at nine institutions between March 2018 and August 2021 and enrolled 50 patients with unresectable or recurrent gastric or esophagogastric junction adenocarcinoma who had previously undergone treatment with fluoropyrimidine-based chemotherapy. Nab-paclitaxel (80 mg/m²) was administered intravenously on days 1, 8, and 15 in combination with ramucirumab (8 mg/kg) on days 1 and 15 of each 28-day cycle. The primary endpoint was progression-free survival. The secondary endpoints included overall survival, response rate, disease control rate, relative dose intensity, and safety. Tumor responses were assessed using the Response Evaluation Criteria in Solid Tumors v1.1.

**Results:**

The median progression-free survival was 5.8 months (95% confidence interval [CI], 4.6–7.0). The median overall survival was 12.5 months (95% CI, 10.6–14.5). Among patients with measurable lesions, the objective response rate was 33.3%, including one case of complete response. The disease control rate was 80%. Grade ≥ 3 neutropenia occurred in 50% of patients, whereas febrile neutropenia occurred in 2%. The relative dose intensity was 86.5% for nab-paclitaxel and 92.8% for ramucirumab.

**Conclusions:**

Reduced-dose nab-paclitaxel combined with ramucirumab demonstrated modest efficacy and favorable tolerability as second-line chemotherapy for advanced gastric cancer. This regimen may represent a practical treatment alternative for patients deemed unsuitable for full-dose nab-paclitaxel due to concerns regarding its toxicity.

**Trial registration:**

This study was registered in the UMIN Clinical Trials Registry on February 14, 2018 (UMIN000031285).

**Supplementary Information:**

The online version contains supplementary material available at https://doi.org/10.1186/s12885-026-16587-x.

## Background

Gastric cancer is the fifth most common malignancy worldwide and the fifth leading cause of cancer-related deaths globally [1]. Chemotherapy remains the standard treatment for unresectable and recurrent advanced gastric cancer (AGC). In recent years, the introduction of targeted therapies, such as immune checkpoint inhibitors, has improved the outcomes of first-line chemotherapy for AGC; however, survival rates remain low [2–4]. In AGC treatment, survival can be improved through appropriate sequential therapy following primary therapy, with appropriate secondary therapy administered after disease progression [5]. However, in clinical practice, a subset of patients with gastric cancer present with advanced age and poor performance status (PS); thus, the selection of suitable sequential therapy should take into account the risks of toxicity and complications [6, 7]. Accordingly, accumulating evidence for secondary therapy that balances sufficient tumor control with acceptable adverse events is essential.

Currently, the selection of second-line treatment depends on HER2 status. For HER2-positive AGC, trastuzumab deruxtecan has recently demonstrated superior overall survival (OS) compared with ramucirumab (RAM) plus paclitaxel (PTX) in the phase III DESTINY-Gastric04 trial, establishing its role as the new second-line standard of care [8]. Conversely, a secondary chemotherapy regimen for AGC comprising combination therapy with PTX and RAM demonstrated high antitumor efficacy in the RAINBOW trial [9]. At the time the present study was conducted (2018–2021), trastuzumab deruxtecan was unavailable as second-line therapy, and PTX + RAM represented the standard second-line regimen, regardless of HER2 status. For HER2-negative AGC, PTX + RAM remains the standard second-line regimen. Although PTX + RAM therapy is tolerable and suitable for many patients with AGC, PTX is insoluble in water, necessitating the use of polyoxyethylene castor oil and alcohol as solvents [10]. This poses risks of hypersensitivity reactions to polyoxyethylene castor oil and alcohol intolerance in some patients [11, 12]. Nanoparticle albumin-bound PTX (nab-PTX) addresses the limitations of PTX by conjugating it with albumin, eliminating the need for solvents [13]. It is used as an alternative taxane agent in various cancers, including breast, pancreatic, and non-small cell lung cancers, with reportedly high antitumor efficacy [14–16]. A phase II trial of nab-paclitaxel (100 mg/m²) plus ramucirumab in patients with advanced gastric cancer demonstrated a favorable outcome, with a progression-free survival of 7.6 months [17]. Consequently, the weekly nab-PTX + RAM (w-nab-PTX + RAM) regimen is widely used in Japan because of its high efficacy and low risk of hypersensitivity reactions to solvents [18]. However, nab-PTX + RAM therapy has been reported to frequently lead to myelosuppression-associated adverse events, such as grade 3 or higher neutropenia, occurring in up to 77% of cases [17]. Consequently, in clinical practice, for patients with advanced age and poor PS, nab-PTX + RAM therapy is often administered at a reduced dose compared with the standard starting dose. However, data on the efficacy of combination therapy using reduced-dose nab-PTX and RAM for AGC, as well as associated adverse events, remain unclear. Accordingly, we conducted this phase II trial to evaluate the efficacy and safety of w-nab-PTX + RAM, in which nab-PTX was administered at a dose of 80 mg/m², as second-line chemotherapy for AGC.

## Methods

### Patients and study design

This multicenter, open-label, single-arm, prospective phase II clinical trial conducted at nine institutions in Japan between March 2018 and August 2021 enrolled 50 patients with unresectable or recurrent gastric or esophagogastric junction adenocarcinoma who had previously undergone treatment with fluoropyrimidine-based chemotherapy. The study protocol was approved by the Institutional Review Board of Yamaguchi University Hospital (approval no. 2017 − 190). The study was conducted in accordance with the tenets of the Declaration of Helsinki and the Clinical Trials Act of Japan. Written informed consent was obtained from all eligible patients prior to enrollment. This study was conducted in accordance with the Japanese Good Clinical Practice (GCP) guidelines, under which surrogate consent by a legal guardian is not permitted for adult patients in interventional oncology trials, except in specific regulatory contexts. The study protocol was registered with the University hospital Medical Information Network Clinical Trials Registry (http://www.umin.ac.jp/ctr/index-j.htm; registration number, UMIN000031285).

### Eligibility criteria

The major inclusion criteria were as follows: (a) histologically confirmed unresectable or recurrent tumors of the stomach or esophagogastric junction; (b) age ≥ 20 years; (c) Eastern Cooperative Oncology Group performance status (ECOG PS) of 0–2; (d) refractory to first-line fluoropyrimidine-based chemotherapy or relapse within 6 months after fluoropyrimidine-based adjuvant therapy; (e) an expected survival of at least 3 months from the time of enrollment; (f) willingness to participate in the study; and (g) adequate organ function based on laboratory tests performed within 14 days before enrollment, defined as white blood cell count ≥ 3,000/mm³ and ≤ 12,000/mm³, absolute neutrophil count ≥ 1,500/mm³, platelet count ≥ 100,000/mm³, hemoglobin ≥ 9.0 g/dL, aspartate transaminase (AST) and alanine transaminase (ALT) levels ≤ 2.5× the upper limit of normal, total bilirubin ≤ 1.50 mg/dL, and serum creatinine ≤ 1.50 mg/dL. Regarding criterion (d), the two criteria were combined based on the established definition of fluoropyrimidine resistance adopted in previous Japanese gastric cancer trials, in which relapse within 6 months after completion of fluoropyrimidine-based adjuvant chemotherapy was considered clinically equivalent to progressive disease during fluoropyrimidine-based first-line chemotherapy, as both populations are presumed to share similar mechanisms of fluoropyrimidine resistance [19].

The major exclusion criteria were as follows: (a) a history of severe drug hypersensitivity, (b) prior treatment with paclitaxel or nanoparticle albumin-bound paclitaxel, (c) active infection, (d) serious comorbidities, including interstitial pneumonia or pulmonary fibrosis, poorly controlled diabetes mellitus, renal failure, or hepatic failure, (e) active synchronous or metachronous malignancy other than gastric cancer; (f) grade ≥ 2 peripheral neuropathy, (g) psychiatric disorders that would impair the patient’s capacity to provide voluntary informed consent or comply with the study protocol, as judged by the attending physician, and (h) any other condition that, in the judgment of the principal investigator or collaborating investigators, would make the patient unsuitable for safe participation in the study.

Regarding criterion (e), active malignancy was defined as a synchronous second primary malignancy or a metachronous second primary malignancy with a disease-free interval of less than 5 years. Lesions equivalent to carcinoma in situ or intramucosal carcinoma that were judged to be curatively treated by local therapy were not considered active malignancies and were not excluded.

### Treatment

The reduced-dose nab-PTX + RAM regimen involved intravenous nab-PTX at 80 mg/m² administered over 30 min on days 1, 8, and 15, in combination with intravenous RAM at 8 mg/kg on days 1 and 15 of each 28-day cycle. Treatment initiation was contingent upon the following criteria: neutrophils *≥* 1,500/mm^3^, leukocytes *≥* 3000/mm^3^, platelets *≥* 100,000, hemoglobin *≥* 9 g/dL, AST and ALT levels *≤* 100 IU/L, total bilirubin *≤* 2 mg/dL, peripheral neuropathy < grade 2, and other non-hematological toxicities < grade 2. Nab-PTX administration was postponed for 1 week if the above criteria were not met, or if any of the following criteria were observed: neutrophils < 500/mm³, platelets < 50,000/mm³, AST or ALT levels exceeding 2.5 times the institutional upper limit, or peripheral neuropathy ≥ grade 2. Dose modification rules were applied independently for each scheduled dosing day. If the criteria were not met on day 1, the entire cycle was postponed for 2 weeks, with day 8 and day 15 doses administered approximately 1 week after the rescheduled day 1 and day 8 doses, respectively. If the criteria were not met on day 8, the dose was postponed by 1 week and administered subsequently, with the day 15 dose administered approximately 1 week thereafter. If the criteria were not met on day 15, the dose was cancelled and the next cycle commenced as scheduled. Patients were offered RAM if they met the following criteria: urinary protein ≤ 1 + on a dipstick test or < 2 g/24 h, systolic blood pressure < 160 mmHg and diastolic blood pressure < 100 mmHg, and RAM-related adverse events < grade 1 at the beginning of the course. If Common Terminology Criteria for Adverse Events (CTCAE) grade ≥ 3 adverse events and grade ≥ 2 thrombocytopenia were observed, the next dose of nab-PTX was reduced from one step to a maximum of two steps (from 80 to 60 to 50 mg/m^2^). If a urinary protein level of 3 + or an adverse event of CTCAE grade ≥ 3 was observed, the next dose of RAM was reduced by one dose level (from 8 mg/kg to 6 mg/kg).

### Evaluations

The primary endpoint of this study was progression-free survival (PFS), defined as the time from enrollment to either disease progression or all-cause mortality. The secondary endpoints included OS, defined as the duration from enrollment to all-cause mortality; response rate; disease control rate; relative dose intensity, defined as the ratio of the actual cumulative dose administered to the planned cumulative dose over the same period, expressed as a percentage; and adverse events. Tumors were evaluated every 8 weeks until disease progression, and objective responses were assessed according to the Response Evaluation Criteria in Solid Tumors (RECIST) guidelines (version 1.1). In patients with non-measurable disease only (*n* = 19), PFS was assessed based on the criteria for progressive disease defined in RECIST version 1.1, including unequivocal progression of non-target lesions, the appearance of new lesions, or clinical deterioration, as determined by the treating physicians. Physical examination and blood tests were mandatory before each course, and white blood cell count, neutrophil count, platelet count, hemoglobin level, AST level, ALT level, total bilirubin level, and urine protein level were measured before drug administration. Adverse events were assessed according to the National Cancer Institute CTCAE, version 4.0.

### Statistical analysis

The threshold PFS was set at 3.8 months and the expected PFS at 6.8 months, based on data from prior second-line trials. Assuming a one-sided alpha level of 5% and 80% power, the required sample size was calculated using the Southwest Oncology Group One-Arm Nonparametric Survival method was 42 patients. Allowing for a small number of ineligible cases, the target sample size was set at 46 patients. PFS and OS were analyzed using the Kaplan–Meier method. All statistical analyses were performed using SPSS version 25.0 (SPSS Corp., Armonk, NY, USA).

## Results

### Patients

**Fig. 1:**
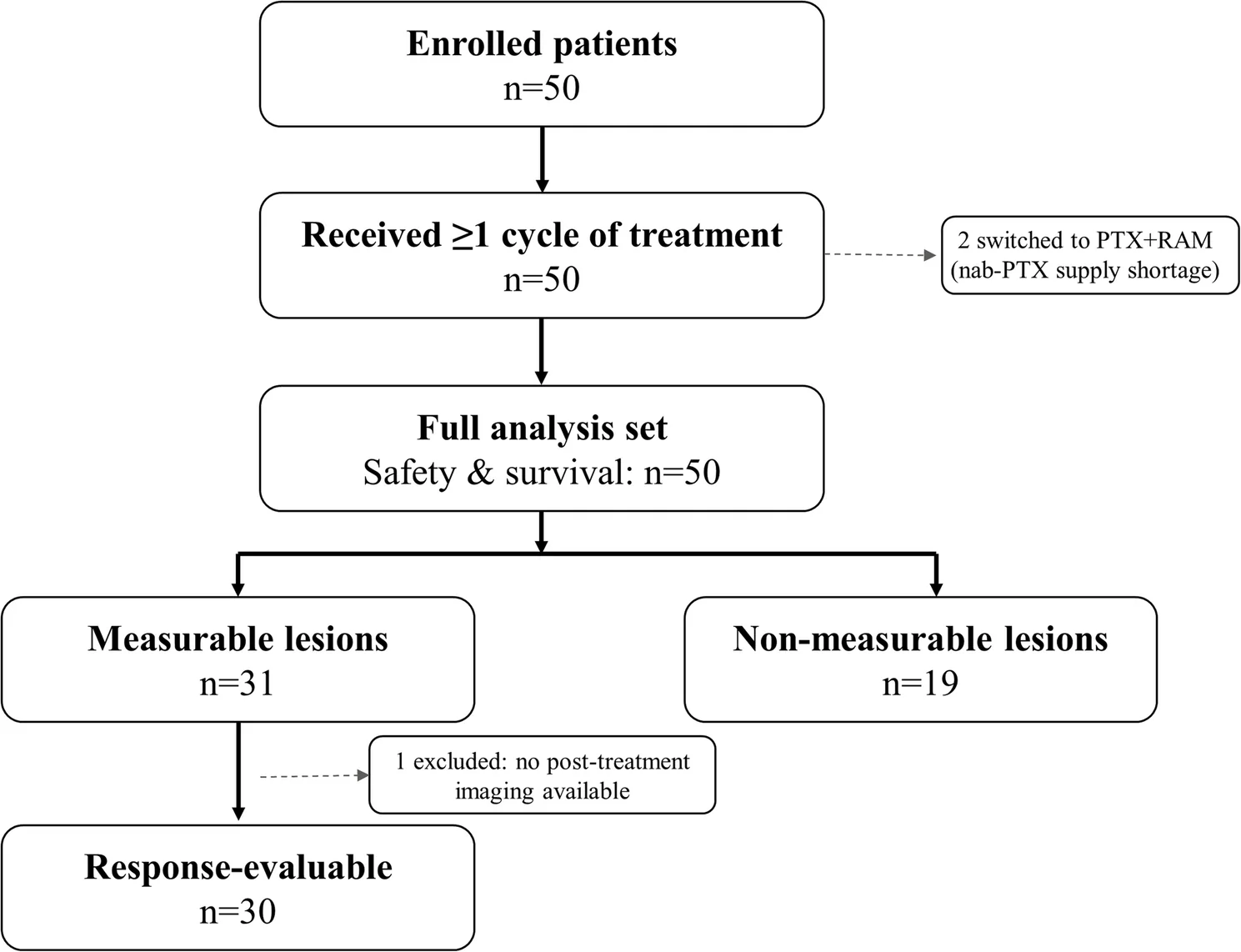
Patient flowchart. All 50 enrolled patients received the study treatment and were included in the full analysis set. Thirty of 31 patients with measurable lesions were evaluable for tumor response. PTX, paclitaxel; nab-PTX, nanoparticle albumin-bound paclitaxel; RAM, ramucirumab

All 50 enrolled patients underwent at least one course of nab-PTX + RAM therapy. Data from all patients were analyzed for safety and survival; however, one patient with evaluable disease was excluded from the objective radiographic response analysis because the post-treatment imaging findings were not available. Owing to restrictions on the supply of nab-PTX by the pharmaceutical company, two patients had to discontinue nab-PTX mid-treatment and were switched to the PTX + RAM regimen. Both patients were followed up for assessment of treatment response and outcomes until disease progression or death, and were included in all efficacy analyses using the intention-to-treat principle. The patient flowchart is shown in Fig. 1.

The characteristics of the 50 eligible patients are presented in Table 1. The patients’ median age was 69 years (range, 31–79), and 33 (66%) were aged ≥ 65 years. Herein, 47 out of 50 patients (94%) were classified as PS0 or PS1; additionally, 40 of the 50 patients had previously received platinum- and fluoropyrimidine-based combination therapy, while the remaining 10 patients had received fluoropyrimidine-based chemotherapy, mainly as adjuvant therapy. The main metastatic sites were lymph nodes (36%), ascites (32%), peritoneal dissemination (32%), and liver metastases (20%), with 31 patients (60%) having measurable disease. Of the 31 patients with evaluable lesions, one did not undergo post-treatment imaging assessment; data from the remaining 30 patients were available for the evaluation of response to treatment.

**Table 1:** Characteristics of eligible patients

Characteristic	*n* = 50
Age, years	
Median (Range)	69 (31–79)
<65	17
≥65	33
Sex	
Male	36
Female	14
Performance status	
0	32
1	15
2	3
Histological type	
Differentiated	18
Undifferentiated	32
HER2 status	
positive	3
negative	47
Previous gastrectomy	
No	23
Yes	27
Prior therapy	
SOX	24
SP	7
XP	4
FOLFOX	3
XELOX	1
S-1 + HER	1
SP + HER	1
S-1	9
Metastatic sites	
Lymph nodes	18
Liver	10
Peritoneum†	16
Ascites†	16
Local recurrence	4
Lung	2
Bone	2
Measurable lesions	
No	19
Yes	31

*Abbreviations: FOLFOX *fluorouracil, leucovorin, and oxaliplatin, *SOX *S-1 plus oxaliplatin, *SP *S-1 plus cisplatin, *XELOX *capecitabine plus oxaliplatin, *XP *capecitabine plus cisplatin, *S-1 + HER *S-1 plus trastuzumab, *SP + HER *S-1 plus cisplatin plus trastuzumab

†Peritoneal dissemination and ascites are recorded as independent metastatic categories and do not necessarily represent the same patients; the identical counts are coincidental

### Response to treatment

**Fig. 2:**
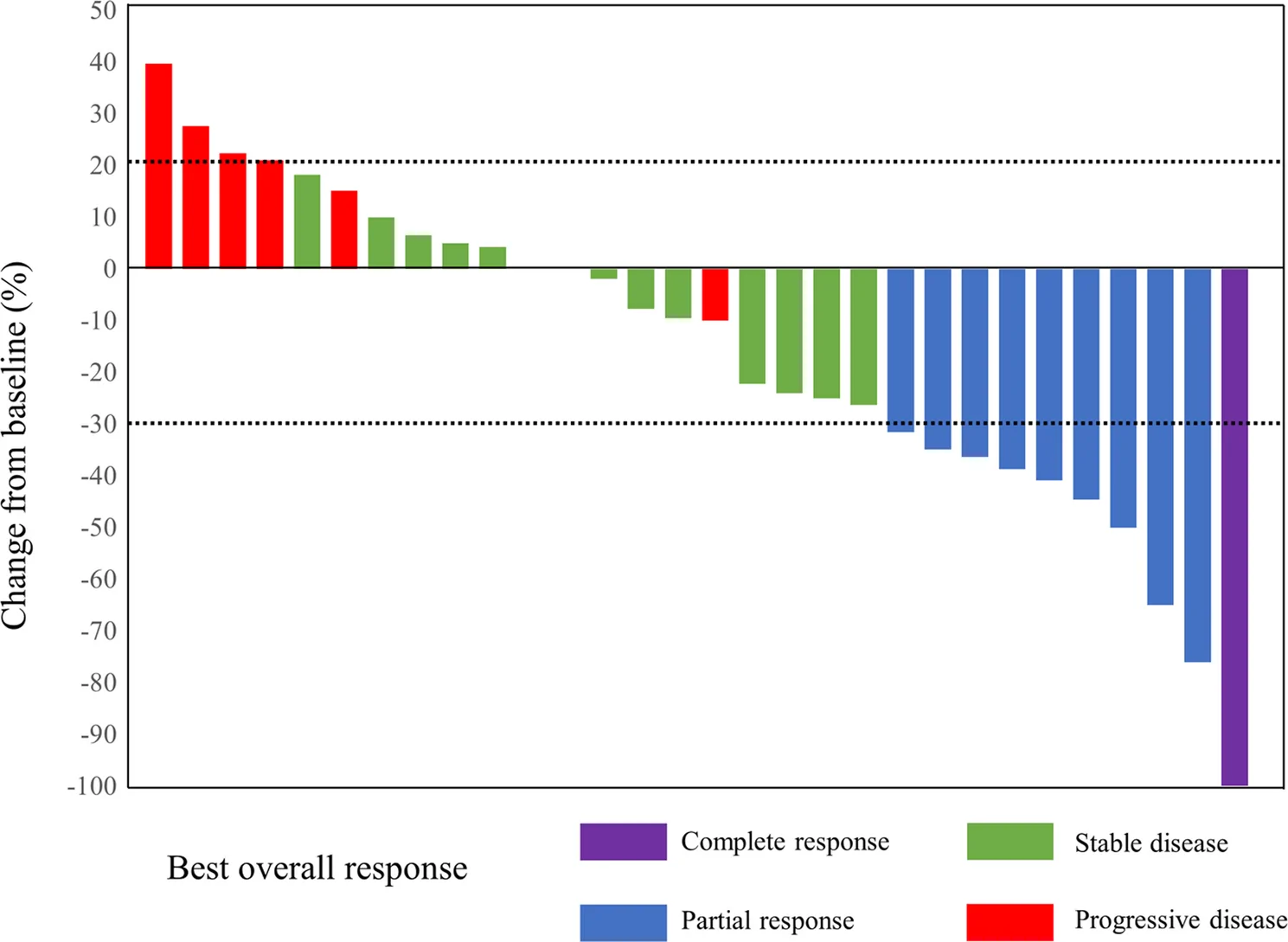
Waterfall plot of best overall response in patients with measurable lesions (*n* = 30). Each bar represents the maximum percentage change from baseline in the sum of target lesion diameters for an individual patient. The upper dashed (+20%) and lower dashed lines (−30%) indicate the Response Evaluation Criteria in Solid Tumors (RECIST) version 1.1 thresholds for progressive disease and partial response, respectively

**Fig. 3:**
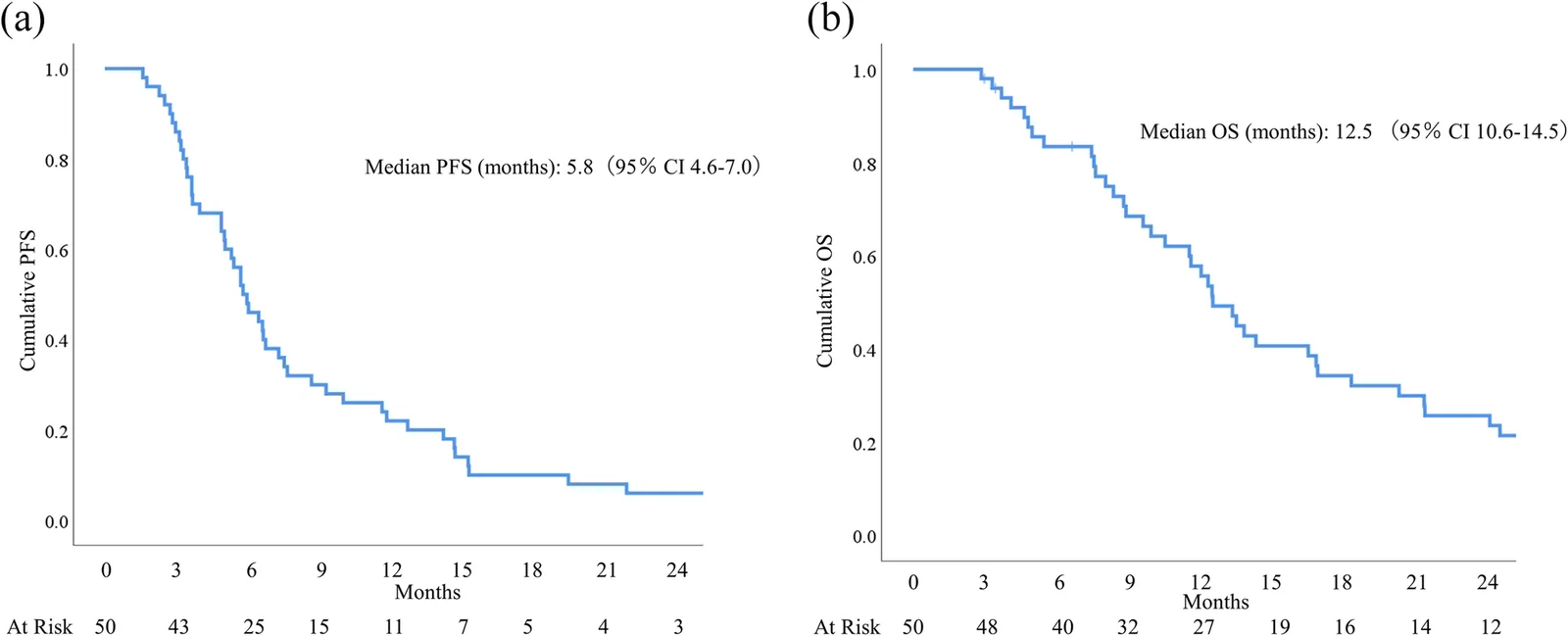
Kaplan–Meier survival curves. **a **Kaplan–Meier curve for progression-free survival (PFS): Median PFS, 5.8 months (95% CI, 4.6–7.0). **b **Kaplan–Meier curve for overall survival (OS): Median OS, 12.5 months (95% CI, 10.6–14.5). Tick marks indicate censored observations

The median follow-up duration was 12.4 months (range, 2.9–60 months). Objective radiographic response was achieved in 10 of 30 patients (33.3%) who were eligible for the evaluation of treatment response. One patient achieved complete response (CR; 3.3%), nine (30%) achieved partial response (PR), and 14 (46.6%) achieved stable disease. The disease control rate—defined as the proportion of patients who had a CR, PR, or stable disease—was 80% (Table 2). A waterfall plot of the best overall response for each patient is shown in Fig. 2. The median PFS was 5.8 months (95% CI 4.6–7.0; Fig. 3a), and the median OS was 12.5 months (95% CI: 10.6–14.5) (Fig. 3b). Among all 50 patients, the PFS was 5.7 months (95% CI, 4.7–6.7) in 31 patients with measurable lesions and 7.3 months (95% CI, 5.1–9.6) in 19 patients without measurable lesions (Additional File 1). Post-progression treatment data were collected for all 50 patients. One patient who achieved CR required no further treatment. Of the remaining 49 patients, 16 (32.7%) received best supportive care only, 31 (63.3%) received nivolumab, one (2.0%) received capecitabine plus oxaliplatin, and one (2.0%) received docetaxel as subsequent therapy.

**Table 2:** Treatment response

Total	*n* = 30
Best overall response	
Complete response	1 (3.3%)
Partial response	9 (30.0%)
Stable disease	14 (46.6%)
Progressive disease	6 (20.0%)
Overall response rate	10 (33.3%)
Disease control rate	24 (80.0%)

### Safety

At least one adverse event of any grade was observed in 48 of 50 patients (96.0%), and grade ≥ 3 adverse events were observed in 34 of 50 patients (68.0%). The median duration of reduced-dose nab-PTX + RAM therapy was 4.2 months. The median relative dose intensities of nab-PTX and RAM were 86.5% and 92.8%, respectively. The most common adverse events of any grade were neutropenia, leukopenia, anemia, fatigue, peripheral neuropathy, and proteinuria. Most adverse events, except for neutropenia, were grade 1 or 2. Neutropenia (*n* = 25, 50%) was the predominant grade ≥ 3 adverse event observed in more than 10% of patients. Febrile neutropenia was observed in one case (2%). A detailed profile of the adverse events is presented in Table 3.

**Table 3:** Adverse events

	Grade, *n*				All grades, *n* (%)	Grade 3, 4 *n* (%)
	1	2	3	4		
Any adverse events						
Hematological						
Neutrophil count decreased	5	12	16	9	42 (84%)	25 (50%)
White blood cell decreased	5	20	12	1	38 (76%)	13 (26%)
Anemia	17	14	3	0	34 (68%)	3 (6%)
Platelet count decreased	17	1	0	0	18 (36%)	0 (0%)
Non-hematological						
Febrile neutropenia	0	0	1	0	1 (2%)	1 (2%)
Peripheral sensory neuropathy	9	14	2	0	25 (50%)	2 (4%)
Fatigue	10	9	6	0	25 (50%)	2 (4%)
Myalgia	0	1	1	0	2 (4%)	1 (2%)
Anorexia	12	8	4	0	24 (48%)	4 (8%)
Stomatitis	3	1	0	0	4 (8%)	0 (0%)
Diarrhea	5	1	0	0	6 (12%)	0 (0%)
Edema limbs	1	2	0	0	3 (6%)	0 (0%)
Nausea	5	3	0	0	8 (16%)	0 (0%)
Vomiting	2	1	0	0	3 (6%)	0 (0%)
Special interest						
Hypertension	8	9	4	0	22 (44%)	5 (10%)
Proteinuria	17	6	1	1	25 (50%)	2 (4%)
Gastrointestinal hemorrhage	0	0	1	0	1 (2%)	1 (2%)
Thromboembolic event	0	0	0	0	0 (0%)	0 (0%)

## Discussion

Several clinical trials have evaluated taxane-based regimens for the treatment of AGC. As a second-line monotherapy, w-PTX has demonstrated a response rate of 16–24%, a PFS of 2.9–3.8 months, and an OS of 7.4–10.9 months [9, 20]. In the ABSOLUTE trial, w-nab-PTX showed favorable outcomes, with a response rate of 33%, a PFS of 5.3 months, and an OS of 11.1 months [21]. In contrast, docetaxel monotherapy yielded a response rate of only 7% and an OS of 5.2 months in the COUGAR-02 trial, highlighting the differences in therapeutic efficacy among taxane agents [22].

Regarding combination therapy with taxanes and RAM, w-PTX + RAM demonstrated a response rate of 28% and a PFS of 4.4 months in the RAINBOW trial, highlighting its superior efficacy over PTX monotherapy [9]. Accordingly, this regimen is currently recommended as the first option for second-line chemotherapy in both Japanese and US clinical guidelines [23, 24]. Furthermore, a phase II study by Bando et al. reported a remarkably high response rate of 55% and a PFS of 7.6 months with nab-PTX + RAM therapy. Additionally, real-world data from Japan indicate that this regimen is frequently used, second only to w-PTX + RAM [17, 18].

However, adverse events associated with taxane-based regimens, particularly myelosuppression and peripheral neuropathy, remain clinically important concerns. Notably, grade ≥ 3 neutropenia reportedly occurs at high frequencies: 29% with w-PTX, 41% with w-nab-PTX, 40.7% with w-PTX + RAM, and as high as 76.7% with w-nab-PTX + RAM [9, 17, 20]. Additionally, febrile neutropenia tends to occur more frequently with w-nab-PTX + RAM (5%) than with other regimens [17]. This may be partly attributable to the higher initial dose of nab-PTX in the w-nab-PTX + RAM regimen (100 mg/m²), which is 25% higher than the PTX-equivalent dose used in the w-PTX + RAM regimen (80 mg/m²) [9, 17]. In addition, previous studies have reported a low relative dose intensity of nab-PTX (61%), indicating that many patients require dose reduction, which is another issue associated with this regimen [17].

The present study was originally designed, prior to the publication of the full-dose w-nab-PTX + RAM trial by Bando et al., to evaluate the clinical efficacy of replacing PTX with nab-PTX at an initial dose of 80 mg/m² in the RAINBOW regimen. Following the publication of Bando et al.’s results, the current study additionally served to investigate the clinical efficacy of a reduced-dose w-nab-PTX + RAM regimen. This dose is equivalent to the PTX dose used at the start of the RAINBOW trial and is expected to mitigate adverse events without substantially compromising treatment intensity.

Herein, the median PFS and OS for the overall cohort were 5.8 months and 12.5 months, respectively, demonstrating relatively favorable outcomes for second-line chemotherapy. The median PFS of 5.8 months observed in this study supported the hypothesis that the reduced-dose regimen achieved the minimum level of clinical activity required for a viable second-line option. Although a direct comparison with the previous full-dose w-nab-PTX + RAM trial was not feasible owing to differences in the eligibility criteria—specifically, the requirement for measurable disease in the previous study—the median PFS among patients with measurable lesions in the present study was 5.8 months, which did not reach the reported PFS of 7.6 months [17]. These findings suggest that, although dose reduction may enhance tolerability, it may be associated with a modest decrease in antitumor efficacy. Regarding post-progression treatment, a substantial proportion of patients (63.3%) received nivolumab as subsequent therapy. This may have contributed to the favorable median OS of 12.5 months observed in this study and should be taken into consideration when interpreting the survival outcomes.

Regarding adverse events, the incidences of grade ≥ 3 neutropenia (50%) and febrile neutropenia (2%) with this regimen were comparable to those observed with w-PTX + RAM and nab-PTX monotherapy. In contrast, hematologic toxicity was lower compared with full-dose w-nab-PTX + RAM, which has a reported incidence of 77% for grade ≥ 3 neutropenia. Considering that 66% of our patients were aged ≥ 65 years, this reduced-dose regimen appears to be a clinically feasible treatment option.

Among regimens developed to improve tolerability by extending the dosing interval of nab-PTX, the B-RAX trial (JACCRO GC-09) evaluated biweekly nab-PTX + RAM in patients who experienced severe adverse events during the initial treatment cycle [25]. Therein, the median PFS was 2.7 months, and grade ≥ 3 neutropenia occurred in 83% of patients. Although a direct comparison is challenging due to differences in patient populations and nab-PTX dosing, the clinical positioning of the B-RAX regimen is similar to that of our study, in the sense that both investigated alternative strategies for patients unable to tolerate standard-dose nab-PTX + RAM.

To our knowledge, this is the first study to report the clinical outcomes of a reduced-dose w-nab-PTX + RAM regimen. This approach may serve as a valuable treatment option for patients who should receive w-nab-PTX rather than w-PTX, such as those with alcohol intolerance but for whom full-dose combination therapy raises safety concerns [11]. Conversely, in patients who can tolerate full-dose therapy, attention must be paid to potential reductions in dose intensity. Nevertheless, a dose of 80 mg/m² is equivalent to the initial PTX dose used in the RAINBOW trial and can be considered an acceptable starting dose [9].

This study has certain limitations. First, this was a single-arm phase II trial with no control group, which limited our ability to draw definitive conclusions regarding the comparative efficacy of the reduced-dose w-nab-PTX + RAM regimen. Although the findings were compared with those of previous studies on full-dose w-nab-PTX + RAM and w-PTX + RAM, differences in patient backgrounds, eligibility criteria, and assessment schedules may have influenced the comparisons. Second, the sample size was relatively small, resulting in wide confidence intervals for the efficacy and safety estimates, which limits the precision of our conclusions. Third, the slow accrual rate—50 patients enrolled over 42 months across 9 institutions, averaging approximately one patient per 10 months per site—and the uneven distribution of enrollment—39 of 50 patients (78%) recruited from three institutions—raise the possibility of selection bias. Patients enrolled at high-volume centers may not be fully representative of the broader population eligible for this regimen, and this limitation should be considered when generalizing our findings. Fourth, the heterogeneity of the study population poses a limitation. Patients refractory to first-line chemotherapy for unresectable or recurrent disease and those experiencing recurrence within 6 months after adjuvant chemotherapy may differ in terms of tumor burden, prior treatment intensity, and prognosis. Because of the limited sample size, we could not perform a reliable subgroup analysis according to these eligibility categories. Therefore, the efficacy of reduced-dose nab-PTX plus RAM in each subgroup should be interpreted with caution. Fifth, tumor response was assessed locally at each institution, without an independent central review by an imaging committee. Although local assessment per the RECIST version 1.1 is standard practice in phase II trials, the absence of an independent radiological review may have introduced variability in the evaluation of response and progression, potentially affecting the reported overall response rate and PFS. Sixth, tolerability was assessed using the CTCAE grading system, which is the standard approach in phase II trials. However, patient-reported outcomes and formal quality-of-life assessments were not incorporated, and therefore the full clinical impact of the safety profile from the patients’ perspective may not be comprehensively reflected. Future studies should incorporate validated patient-reported outcome instruments—such as the European Organisation for Research and Treatment of Cancer Quality of Life Questionnaire Core 30—to enable a more complete evaluation of tolerability. Seventh, owing to a temporary supply shortage of nab-PTX during the study period, two patients discontinued nab-PTX mid-treatment and were switched to PTX. Although these cases represented only a small proportion of the study cohort, the possibility that this deviation influenced overall treatment outcomes cannot be fully excluded.

## Conclusion

Reduced-dose w-nab-PTX + RAM demonstrated favorable tolerability and modest efficacy as a second-line chemotherapy for AGC. This study is the first prospective trial to evaluate a reduced-dose nab-PTX + RAM regimen, which has been widely used in clinical practice in Japan despite the lack of supporting evidence. Our findings provide a practical therapeutic option for patients unsuitable for full-dose nab-PTX-based therapy and offer a clinically relevant foundation for future treatment optimization.

## Supplementary Information

Additional File 1. Kaplan–Meier curve for progression-free survival (PFS). The red and blue lines represent the progression-free survivals of patients with and without measurable lesions, respectively. The median PFS was 5.7 months (95% CI, 4.7–6.7) in patients with measurable lesions and 7.3 months (95% CI, 5.1–9.6) in those without measurable lesions. PFS, progression-free survival; CI, confidence interval.

## Data Availability

The datasets used and/or analyzed during the current study are available from the corresponding author on reasonable request.

## Abbreviations

AGC: Advanced gastric cancer
ALT: Alanine transaminase
AST: Aspartate transaminase
CR: Complete response
CTCAE: Common Terminology Criteria for Adverse Events
Nab-PTX: Nanoparticle albumin-bound PTX
OS: Overall survival
PFS: Progression-free survival
PR: Partial response
PS: Performance status
PTX: Paclitaxel
RAM: Ramucirumab
w-nab-PTX+RA: Weekly nab-PTX+RAM
